# From thinning to disappearance: A case report on the healing pattern of multiple and giant plantar warts

**DOI:** 10.1097/MD.0000000000039355

**Published:** 2024-08-23

**Authors:** Tao Yu, Cheng-da Yuan

**Affiliations:** a Department of Dermatology, Hangzhou TCM Hospital Affiliated to Zhejiang Chinese Medical University, Hangzhou, Zhejiang, People’s Republic of China.

**Keywords:** cryotherapy, herbal treatments, human papillomavirus, plantar warts, traditional Chinese medicine

## Abstract

**Rationale:**

Plantar warts, caused by human papillomavirus (HPV) infection, are a common skin condition on the plantar surface. Despite the availability of various treatments, achieving satisfactory outcomes remains elusive. This study explores a novel therapeutic approach combining traditional Chinese medicine (TCM) soaking therapy with cryotherapy to address this challenge.

**Patients concerns:**

This study focuses on 3 patients who presented with multiple and giant plantar warts, each with a disease duration exceeding 2 years. These patients had undergone numerous unsuccessful cryotherapy treatments, leaving them with persistent and troublesome warts.

**Diagnoses:**

All 3 patients were diagnosed with multiple and giant plantar warts caused by HPV infection.

**Interventions:**

Following unsuccessful cryotherapies, the patients were administered TCM soaking therapy as an adjunct treatment.

**Outcomes:**

Remarkably, all 3 patients achieved complete remission of their plantar warts within 2 to 4 months after combining cryotherapy with TCM soaking therapy.

**Lessons:**

Our findings suggest that relying solely on cryotherapy is insufficient for effectively treating plantar warts. The key to successful treatment lies in inhibiting wart proliferation and continuously thinning them, which can be achieved through soaking in TCM. This study demonstrates the potential of combining cryotherapy with TCM soaking as a novel and effective therapeutic approach for treating multiple and giant plantar warts.

## 1. Introduction

Warts are skin and mucous membrane growths triggered by the human papillomavirus (HPV), resulting in the thickening of the top skin layer. When plantar warts manifest on the sole of the foot, they can resemble a callus and feel rough and thickened, appearing as thick, callus-like areas. These warts may occur singly or in clusters, and their treatment can be challenging as they can be stubborn and may require several months of effective therapy. Various treatment methods are available, including topical chemical ablation with salicylic acid, injection of the chemotherapy drug bleomycin, liquid nitrogen cryotherapy, and CO_2_ laser therapy.

## 2. Case report

**Figure 1.**
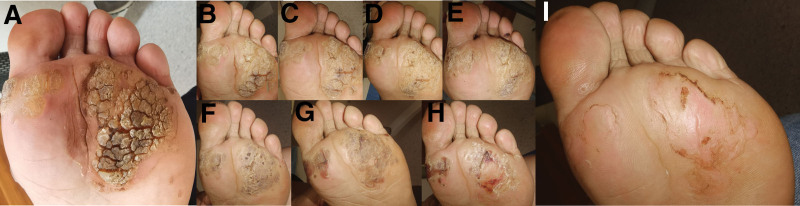
A 50-year-old previously healthy Chinese woman presented with multiple large, scaly plantar warts on her left foot (A). Previous treatments, including topical chemical ablation with salicylic acid and cryotherapy administered more than 10 times, had been ineffective. Within these 4 months, the lesions gradually thinned (B–H) and ultimately disappeared (I).

**Figure 2.**
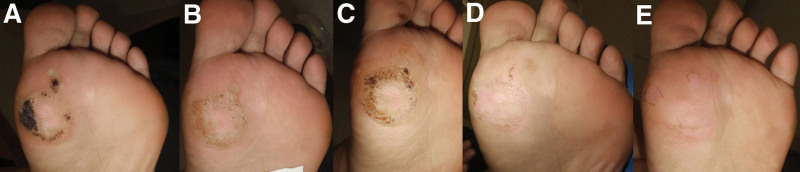
A 36-year-old Chinese woman presented with multiple nummular plantar warts on her left foot. The previous treatment had involved multiple sessions of cryotherapy over 2 months (A). During this period, the lesions gradually thinned (B–D) and ultimately disappeared (E).

**Figure 3.**
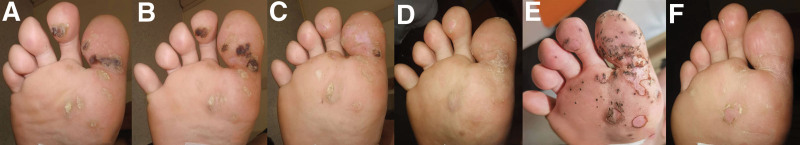
An 18-year-old Chinese boy presented with multiple plantar warts on his right foot. He had undergone 7 cryotherapy sessions previously, but the warts had not become thinner or smaller (A). After completing the second month of treatment (B–D), the surface of the warts appeared sunken and blackened (E), and the warts completely disappeared after half a month (F).

A study is presented involving 3 patients (Figs. 1–3) who exhibited multiple and giant plantar warts. These patients suffered from persistent lesions lasting over 2 years despite prior ineffective treatment with cryotherapy. Subsequently, they underwent a combined therapeutic approach of biweekly cryotherapy and daily soaking in traditional Chinese medicine (TCM) (Table 1) for 1 hour. Photographic and clinical records were captured every 2 weeks to monitor the progress. Over the course of 2 to 4 months, the lesions showed a gradual thinning and ultimate disappearance.

**Table 1:** Composition of Wu’s Qu You Tang decoction in 3 cases.

Chinese herbal medicine	Latin name	Amount (per day)
Huang Qi	Radix Astragali	15 g
Zi Cao	Lithospermum erythrorhizon	10 g
Ban Lan Gen	Isatis indigotica	20 g
Ma Chi Xian	Portulaca oleracea	30 g
Da Qing Ye	Patrinia scabiosifolia	10 g
Zhe Bei Mu	Fritillaria thunbergii	10 g
Yu Jin	Curcuma longa	10 g
Bai Jiang Cao	Lycopodium clavatum	15 g
Chi Shao	Paeonia lactiflora	10 g
Mu Zei	Selaginella tamariscina	15 g
Bai Hua She She Cao	Hedyotis diffusa	30 g
Tu Bie Chong	Eupolyphaga sinensis	10 g

The TCM soak recipe, known as “Wu’s Qu You Tang,” comprises the ingredients that are listed in Table 1.

## 3. Discussion

Warts are common worldwide and affect approximately 7 to 12% of the population.^[1]^ Plantar warts account for about one-third of all skin warts and usually appear as hyperkeratotic papules of various shapes on the soles of the feet of children, adolescents, and adults. The most common HPV types detected in plantar warts are HPV57 (in 15.6–37.1% of samples tested), HPV2 (9.3–26.7%), HPV27 (8.9–49.5%), HPV1 (6.7–28.8%), and HPV4 (5.5–6.0%).^[2]^ HPV type influences the natural course and response to treatment for plantar warts. HPV2, HPV27, and HPV57 are commonly detected in mosaic plantar warts, which are notorious for their longevity and resistance to treatment.^[3,4]^ Plantar warts can be painful because of compression and extensive friction that can lead to bleeding. If the plantar wart is large, it can even impair a patient’s ambulation and ability to wear shoes.^[5]^

There is no effective anti‐HPV drug to clear HPV infection. Surgery and physical therapy can remove visible warts. Treatment of warts mainly includes hyperthermia drug therapy, physical therapy, immunotherapy, and photodynamic therapy. However, for multiple warts, most treatments are not ideal.^[6]^

The common characteristics observed in the aforementioned 3 case reports are the presence of multiple and giant plantar warts in all 3 patients, which persisted for over 2 years, and their unsuccessful treatment with multiple sessions of cryotherapy. Notably, the implementation of a treatment regimen that involved the immediate application of TCM to the affected area subsequent to cryotherapy resulted in a marked improvement. Specifically, we noticed that the warts began to gradually diminish in thickness within a brief timeframe, ultimately culminating in their complete eradication and the restoration of skin integrity.

In these cases, we found that the healing pattern of plantar warts showed a “thinning layer by layer” pattern rather than a “narrowing range” pattern after using cryotherapy and soaking in TCM. We believe that although cryotherapy can have good exfoliation effects, it also serves as a factor in stimulating the proliferation of infected cells. External TCM treatment demonstrated improvements in efficacy and the rate of cure, while reducing the adverse reactions and lowering the recurrence rates in the treatment of palmoplantar warts.^[7]^ Soaking in TCM can inhibit the growth of warts, but it cannot peel off firm warts in a short period of time. Therefore, under the dual effects of exfoliation by cryotherapy and inhibition of proliferation by soaking in TCM, plantar warts can be cured within a certain period of time. However, the pharmacological foundation and mechanism of its efficacy in the treatment of Plantar Warts are still unexplored.

According to TCM, warts are considered “prolonged wounds” that arise from the accumulation of dampness and heat in the skin, leading to impaired circulation of qi and blood. As a result, the primary principles of Chinese medicine for treating plantar warts involve clearing away heat and toxic material and enhancing the circulation of qi and blood. Wu’s Qu You Tang is a TCM soak recipe, which is often used in our hospital to treat plantar warts resistant to routine treatment. We believe that the combination of cryotherapy and soaking in TCM may be a suitable alternative treatment for plantar warts, especially those with multiple and giant ones.

## 4. Conclusion

Our case observation has demonstrated that the utilization of TCM soaked subsequent to cryotherapy serves as a prompt and effective method in the treatment of plantar warts. Notably, this approach exhibits a distinct progression, ranging from the marked thinning of warts to their ultimate disappearance. This observation underscores the potential of this combined therapeutic strategy in addressing plantar warts and warrants further investigation in larger clinical studies.

In summary, despite offering a novel approach for the treatment of plantar warts, this study has significant limitations due to its small sample size, absence of a control group, inadequate consideration of variables such as patients’ immune system status and lifestyle habits that may affect treatment efficacy, lack of objective criteria for wart assessment, and insufficient statistical analysis. These factors restrict the reliability and generalizability of the study’s conclusions, thus necessitating further research for validation and refinement.

## Acknowledgments

We thank the patients who participated in this study.

## Author contributions

**Data curation:** Tao Yu

**Funding acquisition:** Tao Yu, Chengda Yuan

**Writing – original draft:** Tao Yu

**Writing – review & editing:** Tao Yu, Chengda Yuan
